# Effect of Artery Diameter and Velocity and Vein Diameter on Upper Limb Arteriovenous Fistula Outcomes

**DOI:** 10.1155/2022/2158013

**Published:** 2022-03-26

**Authors:** Morwan Bahi

**Affiliations:** Department of Vascular Surgery, Wellington Hospital, Wellington, New Zealand

## Abstract

**Objective:**

Haemodialysis is an important tool for end-stage renal disease (ESRD) patients to correct electrolyte disturbance and improve life quality. This requires a method of long-term vascular access. For many ESRD patients, arteriovenous fistula (AVF) formation is the optimal method of access. AVF success and patency are critical to allow access for haemodialysis. Failure to mature and thrombosis are key factors involved in AVF failure. Our aim is to report outcomes from a single-centre, retrospective study investigating the association between artery and vein measurements as well as intraoperative heparin use on autogenous upper limb AVF outcomes.

**Methods:**

This retrospective study analyses the influence of artery and vein diameter on AVF outcomes over a 12-month period, from 1 January 2019 through 31 December 2019. The key endpoint was AVF patency and functionality at 6 weeks postoperatively.

**Results:**

During our study period, 66 autogenous upper limb AVFs were formed in our tertiary vascular centre. This included 44 radiocephalic, 11 brachiocephalic, and 11 brachiobasilic AVFs. We report an association between minimum vein diameter >2.0 mm and arterial diameter >2.0 mm and AVF success and functionality. Our data did not support intraoperative heparin use as a measure to improve AVF success.

**Conclusion:**

This study suggests a statistically significant association between vein and artery diameter (>2.0 mm) with improved AVF patency at 6 weeks postoperatively. We did not observe benefit from intraoperative heparin use.

## 1. Introduction

End-stage renal disease (ESRD) is an increasingly prevalent pathology worldwide leading to increasing requirement for vascular access for dialysis [1, 2]. Dialysis options include peritoneal dialysis or haemodialysis [1, 2]. The latter requires a form of vascular access, namely, central venous lines or arteriovenous fistulae (AVF) [1, 2]. AVF is a common method of vascular access for ESRD patients, but it is not without its complications. These can include failure to mature or thrombosis.

A number of variables have been analysed as possible predictors for the success and maturation of AVFs. It has been reported that vein and artery diameters play an important role in AVF success [1–5]. A number of trials have also investigated the effect of intraoperative intravenous heparin use for improving AVF patency outcomes [6, 7].

The preoperative work-up before AVF formation varies between clinicians and centres. Some patients will receive a physical assessment before preceding to AVF formation while others will undergo duplex ultrasound vessel-mapping and others will have a venogram performed before the surgery.

In this study, we report 12-month outcomes following upper limb AVF formation in a tertiary vascular centre and consider the influence of multiple factors including vessel diameter and velocity and intraoperative heparin use on AVF outcomes.

## 2. Methods

This retrospective audit was approved by the Wellington Hospital Clinical Audit Group on 17/06/2021 (ID 2021/25) and the need for informed consent was waived.

### 2.1. Study Design

This is a retrospective analysis including all patients undergoing upper limb AVF formation for haemodialysis between 1 January 2019 and 31 December 2019. All patients were referred directly to the vascular surgery department at Wellington Hospital. All data were recorded in an Excel spreadsheet on a secure hospital server. We collected data on age, gender, type of AVF, preoperative imaging, comorbidities, intraoperative heparin use, and outcomes.

### 2.2. Operative Details

All procedures were performed under local or regional anaesthesia. All procedures were performed by 3 vascular surgeons in our tertiary vascular centre. All anastomoses were performed using running proline suture material.

Intraoperative heparin use was based on clinician preference. Where intraoperative heparin was used, the dose was either 2500 or 3000 international units (IU). This was also based on clinician preference.

### 2.3. Pre- and Postoperative Assessment

All patients underwent preoperative duplex ultrasound imaging of the ipsilateral limb. This included measurement of minimum vein diameter and arterial diameter and velocity. Information on vessel quality was not gathered for the purpose of this scan. Tourniquets were used to derive minimum vein diameter.

Patient follow-up was performed typically 1-2 weeks after AVF formation and then again 6 weeks following the procedure by a renal clinical nurse specialist (CNS).

### 2.4. Outcomes and Definitions

The primary outcome measured in this trial was AVF patency and functionality during the follow-up period. This was assessed clinically at 6 weeks postoperatively by the renal CNS. Where the fistula was not functional at 6 weeks, the patient underwent a further duplex ultrasound to clarify the cause of nonfunctionality as either thrombosis or failure to mature.

### 2.5. Statistical Analysis

Statistical analyses were performed using a combination of Excel (Microsoft, Redmond, Washington, USA) and R software (R Foundation for Statistical Computing, Vienna, Austria). Logistical regression was applied to calculate the effect of multiple variables on primary outcome of AVF maturation including minimum vein diameter, artery diameter and velocity, and intraoperative heparin use as well as medical comorbidities including hypertension and diabetes. A *P* value <0.05 was considered statistically significant.

To investigate the effect of vein and artery diameter measurements on AVF maturation and patency, dichotomized cut points were determined using the outcome-based Contal and O'Quigley method [8].

## 3. Results

Between 1 January 2019 and 31 December 2019, 66 autogenous upper limb AVFs were formed in our tertiary vascular centre for the purpose of haemodialysis. These included a combination of 44 radiocephalic (RCF), 11 brachiocephalic (BCF), and 11 brachiobasilic (BBF) fistulae. AVFs were created by 3 vascular surgeons with comparable operative methods. Complete venous and arterial limb ultrasound mapping was completed preoperatively for 100% of patients.

### 3.1. Demographics and Characteristics

The average age was 52.8 years with a slight male predominance of 54.5%. The largest patient group by ethnicity was Māori and Pacific (45.5%) followed by New Zealand European (42.4%). Average BMI was 26.9. Twenty-nine patients (43.9%) were classified as diabetic while 55 (83.3%) exhibited underlying hypertension requiring at least 1 oral antihypertensive agent. The most common aetiology for underlying ESRD was diabetic nephropathy in 24 (36.4%) of the patients followed by glomerulonephritis in 16 (24.2%). A complete summary of demographics, comorbidities, and aetiology is outlined in Table 1.

### 3.2. Preoperative Imaging

All patients completed preoperative duplex ultrasound imaging of the ipsilateral limb. The smallest utilised vein diameter was 2.0 mm (cephalic) with mean minimum vein diameter 2.9 mm. By fistula type, the smallest used vein was 2.0 mm (RCF), 2.3 mm (BCF), and 2.0 mm (BBF). The smallest used arterial diameter was 2.0 mm (radial) with mean 2.86 mm. Correspondingly, smallest arterial diameter by AVF type was 2.0 mm (RCF), 2.8 (BCF), and 3 mm (BBF). The lowest arterial velocity was 45 cm/s (radial) with mean 87.5 cm/s. Details of preoperative imaging are outlined in Table 2.

### 3.3. AVF Outcomes

Sixty-six fistulas were formed. These included 44 RCF, 11 BCF, and 11 BBF. Forty-six (70%) upper limb AVFs matured and became functional at 6 weeks postoperatively. Forty-eight (72.7%) of all AVF formations were classified as the “first access procedure” for the patient. Of the 44 radiocephalic AVFs formed, 30 (68.2%) matured and were functional. This compared with 10 of 11 (90.9%) brachiocephalic and 6 of 11 (54.5%) brachiobasilic AVFs, respectively. Intraoperative heparin was administered during the majority of AVF formation procedures (87.9%). These details are outlined in Table 3.

Twenty AVFs (30%) did not become functional. Key reasons were failure to mature or development of thrombosis.

### 3.4. Regression Analysis

Univariate analyses for all 66 AVFs identified minimum vein diameter >2.0 mm and arterial diameter >2.0 mm to be significant for improved AVF success. On multivariate regression, minimum vein diameter >2.0 mm was associated with 45% higher rate of AVF success (RR 1.45, 95% CI 1.18–2.27, *P*=0.038) while arterial diameter >2.0 mm was associated with 15% higher rate of AVF success (RR 1.15, 95% CI 1.01–3.18, *P*=0.037). Arterial velocity was not associated with improved AVF patency. Heparin use did not result in statistically significant improved AVF patency (RR 1.11, 95% CI 0.54–1.76, *P*=0.200). A complete list is displayed in Table 4.

Regression analysis was repeated for the RCF subgroup. This redemonstrated significant improvement in AVF patency with vein size >2.0 mm on univariate (RR 1.47, 95% CI 0.65–3.67, *P*=0.038) and multivariate regression (RR 1.78, 95% CI 1.16–3.47, *P*=0.028). Statistical significance was also found with artery diameter >2.0 mm on univariate (RR 2.26, 95% CI 1.72–4.36, *P*=0.020) and multivariate regression (RR 2.00, 95% CI 1.01–3.03, *P*=0.034). Arterial velocity and intraoperative heparin use were not associated with improved AVF outcomes, as shown in Table 5.

Statistical significance was not found for any factors for the BCF and BBF subgroup univariate analyses, however noting each group had a limited sample size of 11 (Tables 6 and 7). Multivariate analyses were not possible to a meaningful extent given the small sample size in these subgroups.

## 4. Discussion

This is a single-centre study reporting outcomes following autogenous upper limb AVF formation. We focus our analyses on minimum vein diameter, arterial diameter, and velocity as well as intraoperative heparin use.

Reports in the literature regarding effect of vein and artery diameter on AVF outcomes have varying outcomes [3, 5, 9]. The most consistent findings from existing literature suggest the strongest association is between a minimum vein diameter of 3.0 mm and improved AVF outcomes [3–5, 9]. Other reports suggest no predictive outcome of artery or vein size on AVF outcomes [3, 5]. Some level of discrepancy may be attributed to patient and technical factors including comorbidities and functional factors including use of tourniquet-derived measurements during ultrasound versus no tourniquet. The quality of in-flow vessels (arteries) can also affect outcomes [10].

In our study cohort, we found 2 statistically significant predictors of AVF success. We report a minimum vein diameter of >2.0 mm to be associated with improved AVF patency (RR 1.45, 95% CI 1.18–2.27, *P*=0.038). Equally, we report arterial diameter >2.0 mm to be associated with significant improved outcomes (RR 1.15, 95% CI 1.01–3.18, *P*=0.037). These were reported on multivariate analyses. Subgroup analysis of radiocephalic fistulae found similar significance for vein diameter >2.0 mm (RR 1.78, 95% CI 1.16–3.47, *P*=0.028) and arterial diameter >2.0 mm (RR 2.00, 95% CI 1.01–3.03, *P*=0.034). Although the rate of AVF success by fistula type was highest for brachiocephalic AVF (90.9%; compared with 68.2% for RCF and 54.5% for BBF), our analyses did not show statistical significance of AVF type on outcomes (Table 4). This may have been related to our small sample size.

Intraoperative heparin use has been suggested as a potential mechanism for improving AVF outcomes in the literature [6, 7]. This is thought to be secondary to its anticoagulant activity reducing rates of AVF thrombosis [7]. Our analyses showed no statistical significance for heparin use on AVF success (RR 1.11, 95% CI 0.54–1.76, *P*=0.200).

### 4.1. Limitations, Strengths, and Future Direction

We note a number of limitations in our study. Our data acquisition was retrospective and data came from one centre only. This brings with it geographic bias as well as clinician bias with a small number of surgeons performing all procedures comparing to a multicentre study with larger surgeon variability. Furthermore, the smaller numbers of brachiocephalic and brachiobasilic AVFs limited our ability to perform multivariate analyses for those 2 AVF subgroups. One strength of this study is availability of objective preoperative vessel measurement through duplex ultrasound for 100% of patients.

Further areas of focus include the quality of vessels used for AVF formation. It is well-documented that poor quality artery as an example, a diseased radial artery with microcalcifications can result in poorer AVF maturation rate [10]. Our preoperative duplex ultrasound imaging focuses primarily on measurement of vessel diameter and velocity. We plan in future to study the preoperative quality of vein and artery used for AVF formation and the relation of this on AVF outcomes.

## 5. Conclusion

Formation of autogenous arteriovenous fistulae remains an important method of vascular access for haemodialysis for patients with end-stage renal disease. Key complications following AVF formation are failure to mature and thrombosis. Multiple variables have been suggested to be predictors of AVF success including vessel diameter and flow and other factors such as comorbidities and heparin use. We found a minimum vein and artery diameter >2.0 mm to be statistically significant predictors of AVF success. Arterial velocity, intraoperative heparin use, hypertension, and diabetes were not associated with AVF outcomes. A key limitation of this study is being a retrospective, single-centre study. We aim to continue our study with investigation of the effect of preoperative vessel quality on AVF outcomes next.

## Figures and Tables

**Table 1:** Patient demographics, comorbidities, and ESRD aetiology.

Characteristics	Number (percentage)
Gender	
Male	36 (54.5%)
Female	30 (45.5%)
Age	
Mean (±SD)	52.8 ± 16.9 years
Range	19–79 years
Ethnicity	
Caucasian	28 (42.4%)
Pacific	20 (30.3%)
Māori	10 (15.2%)
Others	8 (12.1%)
BMI	
Mean (±SD)	26.9 ± 4.9
Range	21–38
HTN	
Yes	55 (83.3%)
No	11 (16.7%)
Diabetes	
Yes	29 (43.9%)
No	37 (56.1%)
ESRD cause	
Diabetes	24 (36.4%)
Glomerulonephritis	16 (24.2%)
Others	15 (22.7%)
PCKD	5 (7.6%)
HTN	4 (6.1%)

**Table 2:** Preoperative artery and vein measurements obtained via duplex ultrasound of ipsilateral limb.

Characteristics	Number (percentage)
Minimum vein diameter	
Mean	2.9 mm
Range	2.0–5.3 mm
Maximum vein diameter	
Mean	3.68 mm
Range	2.2–5.5 mm
Artery diameter	
Mean	2.86 mm
Range	2.0–5.6 mm
Artery velocity	
Mean	87.5 cm/s
Range	45–171 cm/s

**Table 3:** AVF procedural factors and outcomes.

Characteristics	Number (percentage)
First access procedure	
Yes	48 (72.7%)
No	18 (27.3%)
Fistula type breakdown	
RCF	44
BCF	11
BBF	11
Intraoperative heparin	
Yes	58 (87.9%)
No	8 (12.1%)
AVF success	
Yes	46 (70%)
No	20 (30%)
Success by AVF type	
RCF	30 (68.2%)
BCF	10 (90.9%)
BBF	6 (54.5%)
Cause for failure	
Failure to mature	12
Thrombosis	8

**Table 4:** Univariate and multivariate analyses between demographics, fistula type, comorbidities, vessel measurements and heparin use, and AVF outcomes.

Variables	Univariate analyses			Multivariate analyses		
	RR ratio	95% CI	*P* value	RR ratio	95% CI	*P* value
Patient age	1.01	(0.97, 1.03)	0.659	1.01	(0.97, 1.05)	0.455
Gender (male)	1.74	(0.60, 5.11)	0.307	2.18	(0.59, 8.49)	0.245
Fistula type (RCF)	1.79	(0.44, 6.94)	0.398	2.06	(0.35, 12.77)	0.420
Fistula type (BCF)	8.33	(1.02, 18.36)	0.080	8.82	(0.89, 21.18)	0.092
Fistula type (BBF)	2.66	(0.44, 1.22)	0.080	2.49	(0.40, 1.18)	0.097
Diabetes	2.99	(0.66, 4.76)	0.120	2.54	(0.90, 3.99)	0.098
Hypertension	1.63	(1.09, 4.90)]	0.440	1.88	(1.54, 4.66)	0.398
Minimum vein diameter	1.80	(1.22, 2.66)	0.044	1.45	(1.18, 2.27)	0.038
Maximum vein diameter	1.51	(0.82, 2.93)	0.197	1.98	(0.69, 6.44)	0.220
Artery diameter	1.57	(0.87, 3.10)	0.016	1.15	(1.01, 3.18)	0.037
Artery velocity	1.01	(0.99, 1.02)	0.385	1.00	(0.97, 1.02)	0.985
Intraoperative heparin	1.35	(0.66, 1.90)	0.2100	1.11	(0.54, 1.76)	0.200

**Table 5:** Univariate and multivariate analyses between demographics and vessel measurements on RCF outcomes.

Variables	Univariate analyses			Multivariate analyses		
	RR ratio	95% CI	*P* value	RR ratio	95% CI	*P* value
Patient age	0.99	(0.94, 1.02)	0.481	0.9890	(0.93, 1.04)	0.692
Gender (male)	2.33	(0.63,8.87)	0.204	1.8487	(0.33, 10.50)	0.474
Minimum vein diameter	1.47	(0.64, 3.67)	0.038	1.7817	(1.16, 3.46)	0.027
Maximum vein diameter	1.87	(0.86, 4.44)	0.128	2.0629	(0.59, 8.76)	0.281
Artery diameter	2.26	(1.71, 4.35)	0.020	1.5271	(0.38, 8.13)	0.571
Artery velocity	1.01	(0.98, 1.03)	0.442	2.0041	(1.01, 3.03)	0.034

**Table 6:** Univariate analyses between demographics and vessel measurements on BCF outcomes.

Variables	Univariate analyses		
	RR ratio	95% confidence interval for RR	*P* value
Patient age	1.12	(1.01, 1.34)	0.080
Gender (male)	4.00	(0.31, 107.04)	0.317
Minimum vein diameter	1.27	(0.27, 7.10)	0.753
Maximum vein diameter	0.86	(0.19, 3.67)	0.837
Artery diameter	1.25	(0.41, 4.63)	0.692
Artery velocity	1.00	(0.95, 1.05)	0.913

**Table 7:** Univariate analyses between demographics and vessel measurements on BBF outcomes.

Variables	Univariate analyses		
	RR ratio	95% confidence interval for RR	*P* value
Patient age	1.02	(0.88, 1.16)	0.769
Gender (male)	0.68	(0.45, 2.99)	0.998
Minimum vein diameter	1.02	(0.03, 2.00)	0.989
Maximum vein diameter	2.03	(0.01, 50.14)	0.592
Artery diameter	1.05	(0.06, 2.46)	0.970
Artery velocity	0.99	(0.88, 1.09)	0.897

## Data Availability

The data used to support the findings of this study are available from the author upon request.
